# Circadian rhythm dysfunction in glaucoma: A hypothesis

**DOI:** 10.1186/1740-3391-6-1

**Published:** 2008-01-10

**Authors:** Girardin Jean-Louis, Ferdinand Zizi, Douglas R Lazzaro, Arthur H Wolintz

**Affiliations:** 1 Department of Ophthalmology, SUNY Downstate Medical Center, New York, USA; 2 Sleep Disorders Center, Department of Neurology, SUNY Downstate Medical Center, New York, USA; 3 Brooklyn Research Foundation on Minority Health, Kingsbrook Jewish Medical Center, New York, USA; 4 Brooklyn Center for Health Disparities, SUNY Downstate Medical Center, New York, USA

## Abstract

The absence of circadian zeitgebers in the social environment causes circadian misalignment, which is often associated with sleep disturbances. Circadian misalignment, defined as a mismatch between the sleep-wake cycle and the timing of the circadian system, can occur either because of inadequate exposure to the light-dark cycle, the most important synchronizer of the circadian system, or reduction in light transmission resulting from ophthalmic diseases (e.g., senile miosis, cataract, diabetic retinopathy, macular degeneration, retinitis pigmentosa, and glaucoma). We propose that glaucoma may be the primary ocular disease that directly compromises photic input to the circadian time-keeping system because of inherent ganglion cell death. Glaucomatous damage to the ganglion cell layer might be particularly harmful to melanopsin. According to histologic and circadian data, a subset of intrinsically photoresponsive retinal ganglion cells, expressing melanopsin and cryptochromes, entrain the endogenous circadian system via transduction of photic input to the thalamus, projecting either to the suprachiasmatic nucleus or the lateral geniculate nucleus. Glaucoma provides a unique opportunity to explore whether in fact light transmission to the circadian system is compromised as a result of ganglion cell loss.

## Background

### Light in human physiology

Numerous experimental studies have convincingly shown that light is the most important synchronizer of circadian rhythms [1]. On a daily basis, light sets and resets the timing of the circadian time-keeping system, to ensure its proper functioning. Consistent with established phase response curves, light exposure early in the morning resets the circadian system to adjust for its propensity to phase-delay, and light exposure in the evening is necessary to adjust for phase-advances in the master clock [2-6].

In the absence of the entraining effects of the light-dark cycle, the circadian system free-runs [7]. According to time-isolation studies, the circadian system can gradually shift and may even become completely desynchronized with respect to external environmental cycles [7,8]. Thus, failure to achieve such necessary adjustments in the timing of the circadian system, either because of deprivation of light cues, knowledge of time, or societal pressures [9,10], could lead to circadian misalignment (see below), which could engender circadian disorders. Paradoxically, the ability of organisms to free-run is considered an advantage, as it allows them to maintain a stable phase relation with the environmental cycles and/or to adapt to seasonal variations in day length [11].

### Ocular pathology and light resistance

Research conducted with visually intact adults has shown that the photosensory brightness-sensing system provides direct photic input to the suprachiasmatic nuclei (SCN, the master circadian clock in mammals), using pathways that are distinct from the standard retinal radiations to the visual system [12-14]. Studies distinguish between two entraining photic pathways. The primary pathway uses the retinohypothalamic tract that transmits photic cues to the SCN and the pineal gland [15,16]. The secondary pathway referred to as the geniculohypothalamic tract stems from the intergeniculate leaflet of the lateral hypothalamus [17-19].

Much of the literature in human chronobiology has focused on studies of aging volunteers in good health. Little has been done to study effects of age-related anatomic and physiologic changes on the photic system, but it is known that important structural SCN alterations as well retinal diseases affect circadian rhythmicity. Research has demonstrated both cellular and structural alterations of the SCN [20,21]. Indeed, neuropathologic studies have revealed selective deterioration of the SCN cell group (i.e., arginine-vasopressin cells) [20,22,23]. Animal studies show age-associated reduction in the amplitude of the rhythm of mRNAs for arginine-vasopressin [24]. In addition, investigations of the expression of mPer1 mRNA and mPer2 mRNA suggest young mice have a stable and well-synchronized circadian rest-activity cycle, whereas older mice display rhythms that are less stable and phase-advanced [25]. These results are consistent with the finding that hormonal secretions (e.g., melatonin, prolactin, TSH, and cortisol) of older adults occur earlier in the day than for younger persons [26-29]. Similarly, body temperature has also shown age-related phase advances [30,31].

Research also shows that a number of ophthalmic factors are associated with age-related reduction in light transmission. One such factor is senile miosis, a condition characterized by a reduction in the diameter of the pupil reducing light transmission to the retina [32]. Opacification of the ocular lens can reduce light transmission by as much as 99%, at which point cataract surgery may become necessary [33]. The finding that loss of retinal ganglion cells occurs in glaucoma [34] may yet be the most important observation concerning circadian human physiology. In severe cases, patients with glaucoma can lose up to 95% of their retinal ganglion cells [35,36]. Ganglion cell death may have direct and adverse effects on the circadian timing system (see 'Glaucoma and Melanopsin Cells').

Age-related reduction in light responsivity has also been demonstrated in animal research. One study showed that circadian phase responsivity to light may be 20 times as great in young hamsters as in old hamsters [37]. Furthermore, older rats seem to need brighter light in order to achieve a desired amplitude of the activity rhythm [38,39]. It is not entirely clear how age-related ocular and neural changes compromise the effectiveness of ambient illumination in maintaining circadian entrainment in humans. Based on observational data from the laboratory and in the home environment, older adults exhibit advanced circadian phases of sleep, cortisol, and aMT6s onset [29]. More research is needed to ascertain whether in fact older individuals develop light resistance because of ocular/optic diseases. It's also important to determine the best light stimulus for optimal circadian entrainment among adults with ophthalmic diseases.

The concept of light resistance refers to a reduction in the expected physiologic or behavioral response to light exposure. Older adults might be particularly vulnerable because of a greater likelihood of experiencing visual impairment [40,41]. Light resistance could also be defined as a reduction in light suppression of melatonin. It is hypothesized that individuals showing light resistance would require a greater amount of illumination to normalize circadian synchronization, thereby improving circadian-related functions. An average amount of illumination, as normally observed in the home environment of the elderly [42-44] would, therefore, be inadequate to prevent circadian misalignment [5,8].

It is noteworthy that a study of experimental bright light treatments showed improvement of circadian rhythm functions only among patients with intact vision, but not among visually impaired patients [45]. Investigators at the University of California, San Diego found that some older adults showed resistance to light treatments, and as many as 30% of them were characterized by circadian parameters outside the range of healthy young adults [46]. Work done by the same investigative group also revealed that one hour of bright green light (1,200 Lux) had no significant antidepressant effect in depressed elders [47]. Negative findings regarding benefits of bright light treatments might be explained by inadequate compensation for deficits in the peripheral visual system associated with aging.

Failure to achieve desirable results in studies of light therapy might also be due to inadequate methodological control for ophthalmic diseases. A recent study conducted among adults ages 50–85 years showed that adjusting for effects of ophthalmic factors reduced the magnitude and strength of the correlations between ambient illumination level and mood [48]. Evidence also suggests that the timing of endogenous melatonin is earlier among individuals with elevated intraocular pressure and large optic cup-to-disk ratios, two common indices of glaucoma [49].

### Ophthalmic diseases and circadian rhythms

No conclusive evidence supports a cause-and-effect relationship between varying ophthalmic diseases and circadian-rhythm functions, although it is well established that in cases where blindness ensues the affected individual experiences poorly entrained circadian rhythms, along with sleep disturbances and depressed moods [40,50,51]. Ophthalmic diseases that might affect photic input to the circadian system include cataract, diabetic retinopathy, macular degeneration, retinitis pigmentosa, optic nerve atrophy, and glaucoma.

Plausibly, cataract, defined as opacity of the crystalline lens of the eye, does not diminish light input significantly unless the disease is far advanced. Such might be the case in densely Brunescent or Morgagnian cataracts [52]. Diabetic retinopathy, resulting from diabetes complication, can cause severe vision loss or even blindness [53]. Diabetic retinal disease varies in severity; hence, light input to the circadian system might be affected differently based on the disease process. Certainly, an end-stage scarred retina from diabetic proliferative disease would be transmitting less light input centrally. Further, a pan-retinal lasered eye in the setting of diabetic disease has lost function in many discrete areas, which would also reduce light stimuli reaching the circadian system. Age-related macular degeneration is a disease process affecting various layers in the deep retina with possible scarring and loss of transmission of light stimuli [54]. This is evident from examination of geographic atrophy and disciform scarring seen in end-stage exudative disease. Retinitis pigmentosa is recognized by a progressive degeneration of the rods, leading to night blindness and loss of peripheral visual field [55]. Clinical manifestations of this disease include pigment deposition in the retina and attenuation of retinal blood vessels. Finally, any optic nerve disease that is characterized by a reduced axonal number would have a relative diminution of transmitted stimuli compared to an eye with a normal optic nerve.

It is of great interest to determine how these ophthalmic diseases each affect the circadian timing system. Evidently, since all these diseases lead to visual impairment and worse yet blindness, they indirectly provoke physical inactivity, which may cascade into sleep problems and daytime sleepiness. Likewise, afflicted individuals would have less opportunity for exposure to bright light exposure, which may cause circadian rhythm dysfunctions [44,56]. By contrast to the aforementioned ocular diseases, the effects of glaucoma on the circadian timing system might be twofold: 1) a direct impact through degeneration of retinal ganglion cells and/or ocular ischemia and reperfusion damage and 2) an indirect impact through social isolation due to blindness, as is the case for other ophthalmic diseases.

Available data point to glaucoma as the main ocular disease that could directly compromise light input to the circadian system. Glaucoma is an ocular degenerative disease that affects ganglion cells, eventually causing optic nerve dysfunction by way of axonal loss [57,58]. Reduced axonal stimulation to the central visual pathways likely diminishes light input to the circadian system. This is supported by recent evidence that melanopsin, which is found in retinal ganglion cells, is a major photopigment involved in circadian entrainment [59-61].

There are two other lines of empirical studies favoring glaucoma as an ocular disease potentially affecting the circadian system. Ample evidence shows that intraocular pressure, the accepted marker of glaucoma severity, exhibits circadian rhythmicity [62-64], which is affected by sleep [65]. Research conducted to test the vascular theory of glaucoma has also yielded supportive evidence linking glaucoma to the circadian system [66]. Using novel hemodynamic methods [67], investigators have shown that reduced retinal perfusion pressure, commonly observed in glaucoma, has a negative effect on the optic nerve head [66,68-70], which might be particularly harmful when occurring at night [71]. Some suggest that optic nerve vascular dysregulation might be secondary to sleep apnea-induced arterial hypertension and arteriosclerosis [72,73]. There is also preliminary data indicating that reduced retinal blood flow is independently associated with the timing and mesor of aMT6s, a melatonin metabolite [74].

### Circadian misalignment

Circadian misalignment refers to a mismatch between individuals' desired bedtime and the timing of their circadian system [75]. This is often observed among shift workers and individuals suffering from jet-lag [76]. This occurs mostly when individuals attempt to initiate sleep at suboptimal times during their circadian cycles; consequently, they may experience sleep disturbances (e.g., difficulty initiating sleep, difficulty maintaining sleep, or early morning awakenings). Similarly, when the circadian system becomes abnormally advanced or delayed with respect to the desired bedtime, even individuals sleeping at optimal times might experience similar sleep disturbances. This phenomenon is widely recognized as advanced-sleep-phase syndrome or delayed-sleep-phase syndrome [77-79].

#### Circadian misalignment in glaucoma: a hypothesis

In adults with healthy eyes, bright light treatment of specified intensity, wavelength, and duration is very effective in treating circadian disorders. Among individuals with varying ocular diseases, the intensity of daylight exposure or artificial light necessary for optimal circadian entrainment has not been systematically established. Moreover, among visually impaired individuals (as may be caused by untreated glaucoma), the consequences of circadian misalignment have not been empirically assessed, but available data suggest that visual and/or photic impairment might cause dampened endogenous rhythms, depression, and sleep disturbances [41,80-83].

Individuals who are blind generally experience circadian rhythm disturbances [40,50,84], but circadian desynchronization might be observable only among those with optic diseases. A study investigating adolescents and young adults ages 12–20 years from the Missouri School for the Blind indicated that patients with optic diseases showed significantly greater circadian dysfunction (e.g., more daytime napping and variable timing of awakening), relative to those without such diseases [85]. Another study investigating circadian rhythms of melatonin and body temperature of 15 patients with No Light Perception found that 9 of those patients maintained circadian synchronization, although the phase angle of entrainment was atypical [86].

These findings are important in the sense that they support the notion that the traditional visual photoreceptors (rods and cones), which are necessary to form visual images, do not appear to have a primary role in circadian entrainment. Rather, light-regulated functions – entrainment of the circadian pacemaker, suppression of activity and melatonin rhythms [87,88], and modulation of pupillary reflex [89,90] – involve specialized signal transduction mechanisms of intrinsically photosensitive retinal ganglion cells [91-93]. These cells are believed to harbor melanopsin, the primary photopigment in the synchronization of circadian rhythms [60,94,95].

#### Role of melanopsin in circadian rhythms

**Figure 1:**
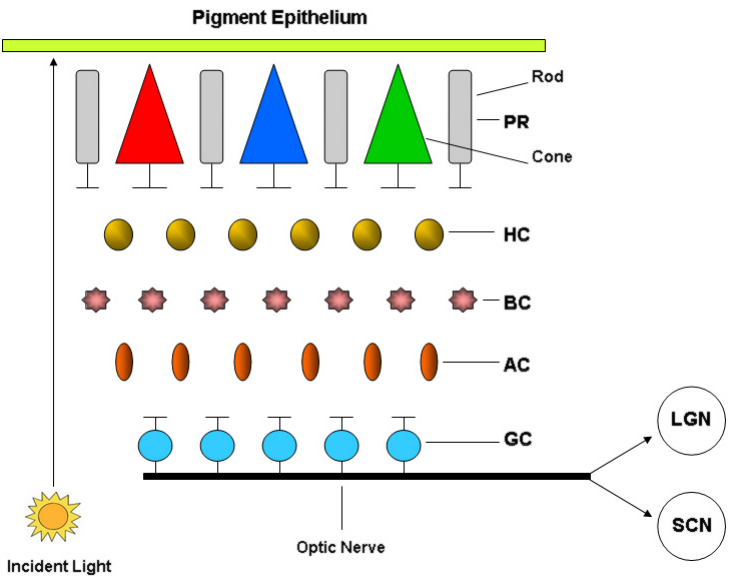
**Illustration of ocular photic transmission pathway**. As light enters the eyes, it is absorbed by photopigments in either the rods or cones in the photoreceptive field (PR), which convert it into a voltage signal. The signal triggers a cascade of synaptic activities through activation of second-order neurons: horizontal cell (HC), bipolar cells (BC), and amacrine cells (AC), some having excitatory action and others inhibitory. The ganglion cells, referred to as third-order neurons, then carry photic input all the way to the thalamus, projecting either to the suprachiasmatic nuclei (SCN) or the lateral geniculate nucleus (LGN). A subset of intrinsically photoresponsive retinal ganglion cells (ipRGCs), expressing melanopsin and cryptochromes, feed the circadian system.

Melanopsin is an opsin-like protein whose coding messenger RNA is found in a subset of retinal ganglion cells. These cells transduce environmental light cues and project to the SCN via the retinohypothalamic tract [59]. Evidence suggests that melanopsin cells have lower sensitivity and spatiotemporal resolution than rods and cones, and they inherently encode ambient light intensity [59,60]. Retinal phototransduction to the SCN is governed largely by melanopsin [59,60], although some evidence continues to point to cryptochromes as key contributors in the generation of circadian rhythms [59,96]. Melanopsin-containing retinal ganglion cells also receive synaptic inputs from rod and cone photoreceptors (see Figure 1) [97,98]. Genetic ablation studies indicate that photic input is abolished only when both melanopsin cells and rod/cone photoreceptors are disabled [90,97]. Conceivably, retinal ganglion cells respond to light input via a melanopsin-based signaling pathway and via a synaptic cascade triggered by rod and cone photoreceptors.

The SCN transmit the retinal signal indirectly to pinealocytes, which in turn transform it into a neuroendocrine message that synchronizes important physiologic and behavioral processes to a 24-hour day [8,88]. Notwithstanding its importance in circadian rhythmicity, evidence is lacking with regard to melanopsin's absorbance spectrum, which may be shorter than spectra established from behavioral and electrophysiological studies. One estimate suggested that melanopsin was most efficiently excited by blue light (420–440 nm) [99].

The melanopsin-containing retinal ganglion cells are widely distributed in the peripheral retina, with dendrites spreading throughout the retina, as might be expected of an illumination-measurement system. Using a melanopsin antibody and/or cRNA probes in combination with PACAP immunostaining, colocalization studies indicated that melanopsin is located exclusively in the PACAP-containing retinal ganglion cells on the surface of soma and dendrites [100]. They are expressed in the majority of retinal ganglion cells projecting to the SCN, ventral subparaventricular zone, and ventrolateral preoptic nucleus as well as in a subpopulation of retinal ganglion cells innervating the pretectal area and the intergeniculate leaflet, through which light has secondary access to the SCN.

It is estimated that two-thirds of retinal ganglion cells containing melanopsin transcript project to the SCN and contralateral pretectal area, and one-fifth projects to the ipsilateral intergeniculate leaflet [101]. According to one study mice that are melanopsin deficient seemed to exhibit complete loss of photo-entrainment of the SCN [102]. Melanopsin activates the photoreceptor G-protein, transducing, in a light-dependent manner, and it is not concentrated in the macula [99]. Accordingly, the peripheral ganglion cell damage noted in glaucoma might be particularly harmful to the retinohypothalamic melanopsin cells.

The evidence summarized above supports the notion that, in mammals, the functionality of photoreception is twofold: one associated with creating a detailed visual image of the environment and another involving the photic regulation of temporal or circadian biology [103]. While understanding the intricacies of these associations await further investigation, there is a body of research evidencing that abnormalities of the circadian timing system may be directly related to depressive symptoms and circadian dysregulations [5,104]. Misalignment of the circadian pacemaker, expressed either as delayed sleep phase syndrome or advanced sleep phase syndrome, or depression and insomnia may be caused by inadequate exposure to day/light cycles or by ophthalmic disease that reduce light signal transmission, or both.

#### Glaucoma and melanopsin cells

One might conjecture that glaucomatous retinopathies could predispose individuals to develop circadian misalignment, since ganglion cell damage in glaucoma might result in melanopsin cell death. Glaucoma represents the principal ophthalmic disorder to explore whether in fact light transmission to the SCN is compromised as a result of melanopsin cell loss. In fact, a recent study using in-situ hybridization and immunocytochemistry to study 17 human eyes and hypothalami containing the SCN, found an even distribution of melanopsin in the retina [105]. Equally important were the finding of reduced retinal ganglion cells in two patients with glaucoma and the observation that melanopsin was conserved in retinae of blind patients with degeneration of the outer and/or inner layers. Evidently, these findings should be replicated in large-scale studies before definitive conclusions can be reached. Likewise, future comparative studies should investigate whether individuals with other retinal diseases (e.g., retinitis pigmentosa) would also show reduced retinal ganglion cells.

We should note that data from animal studies are much less consistent. In the macaque monkey with experimentally induced glaucoma, loss of retinal ganglion cells results in the abolishment of the scotopic threshold response using corneal flash electroretinograms [106]. Investigators made a similar observation after they induced retinal ganglion cell death through transection of the optic nerve of rats [107]. Even when mutant mice were able to generate scotopic threshold response, they were unable to maintain photoentrainment without the presence of retinal ganglion cells [108]. On balance, another animal study suggests that melanopsin-expressing retinal ganglion cells might be less susceptible to death after induction of chronic ocular hypertension [109]. Seemingly, loss of retinal ganglion cells leads to responses that are species specific.

**Figure 2:**
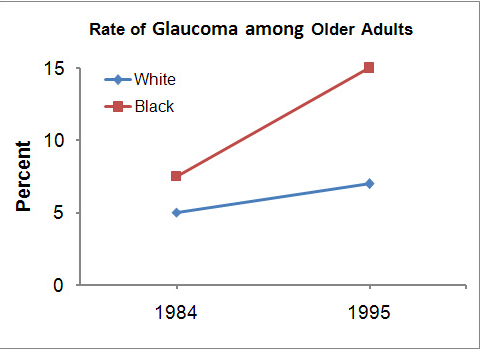
**Percentage of older adults with glaucoma**. Notice that the ethnic difference in glaucoma for both men and women widened between 1984 and 1995. Data originated from the U.S. Centers for Disease Control and Prevention.

Considering the relatively high prevalence of ophthalmic diseases [57,58,110] in aging Americans, it is important to study the link of ophthalmic dysfunction to circadian rhythm dysfunctions [57,58,110]. If indeed impairment of the photic pathway is linked to certain type of sleep disturbances and depression, individuals from the Black race might be at higher risks for depressed mood and disturbed sleep related to aging eyes. Glaucoma, for instance, is more prevalent among Blacks (see Figure 2) [58,110], and ambient light data have shown reduced illumination among Black men and women, compared to their White counterparts [44]. This may lead to renewed effort for early detection of glaucoma, as treatment will not only halt further optic nerve damage but also may help preserve circadian photo-entrainment.

## Competing interests

The author(s) declare that they have no competing interests.

## Authors' contributions

GJL formulated initial ideas for review and drafted the manuscript. FZ performed literature search and reviewed the manuscript. AW assisted in the drafting of the manuscript. DL assisted in the drafting of the manuscript. All authors read and approved the final manuscript.

## References

[B1] Johnson CH, Johnson CH (1990). An Atlas of Phase Response Curves for Circadian and Circatidal Rhythms.

[B2] Czeisler CA, Johnson MP, Duffy JF, Brown EN, Ronda JM, Kronauer RE (1990). Exposure to bright light and darkness to treat physiologic maladaptation to night work [see comments]. Scand J Work Environ Health.

[B3] Lewy AJ, Wehr TA, Goodwin FK, Newsome DA, Markey SP (1997). Light suppresses melatonin secretion in humans. J Am Geriatr Soc.

[B4] Lewy AJ (1996). Effects of light on human melatonin production and the human circadian system. Brain Res.

[B5] Lewy AJ, Sack RL, Miller LS, Hoban TM (1991). Antidepressant and circadian phase-shifting effects of light. J Pineal Res.

[B6] Lewy AJ, Sack RL, Singer CM (1987). Immediate and delayed effects of bright light on human melatonin production: shifting "dawn" and "dusk" shifts the dim light melatonin onset (DLMO). Horm Metab Res.

[B7] Czeisler CA, Duffy JF, Shanahan TL, Brown EN, Mitchell JF, Rimmer DW, Ronda JM, Silva EJ, Allan JS, Emens JS, Dijk DJ, Kronauer RE (1999). Stability, precision, and near-24-hour period of the human circadian pacemaker .. Science.

[B8] Czeisler CA, Richardson GS, Zimmerman JC, Moore-Ede MC, Weitzman ED (1980). Entrainment of human circadian rhythms by light-dark cycles: a reassessment. Photochem Photobiol.

[B9] Elmore SK, Betrus PA, Burr R (1992). Light, social zeitgebers, and the sleep-wake cycle in the entrainment of human circadian rhythms. J Pineal Res.

[B10] Mistlberger RE, Skene DJ (2005). Nonphotic entrainment in humans?. J Biol Rhythms.

[B11] Paranjpe DA, Sharma VK (2005). Evolution of temporal order in living organisms. J Circadian Rhythms.

[B12] Czeisler CA, Kronauer RE, Allan JS, Duffy JF, Jewett ME, Brown EN, Ronda JM (1989). Bright light induction of strong (type 0) resetting of the human circadian pacemaker. Science.

[B13] Czeisler CA, Allan JS, Strogatz SH, Ronda JM, Sánchez R, Ríos CD, Freitag WO, Richardson GS, Kronauer RE (1986). Bright light resets the human circadian pacemaker independent of the timing of the sleep-wake cycle. Science.

[B14] Reme CE, Wirz-Justice A, Terman M (1991). The visual input stage of the mammalian circadian pacemaking system: I. Is there a clock in the mammalian eye?. J Biol Rhythms.

[B15] Cahill GM, Menaker M (1987). Kynurenic acid blocks suprachiasmatic nucleus responses to optic nerve stimulation. Brain Res.

[B16] Cahill GM, Menaker M (1989). Effects of excitatory amino acid receptor antagonists and agonists on suprachiasmatic nucleus responses to retinohypothalamic tract volleys. Brain Res.

[B17] Ohi K, Takashima M, Nishikawa T, Takahashi K (1991). N-methyl-D-aspartate receptor participates in neuronal transmission of photic information through the retinohypothalamic tract. Neuroendocrinology.

[B18] Rietveld WJ (1992). Neurotransmitters and the pharmacology of the suprachiasmatic nuclei. Pharmacol Ther.

[B19] Buijs RM, Kalsbeek A, Romijn HJ, Pennartz CMA, Mirmiran M, Buijs RM, Kalsbeek A, Romijn HJ, Pennartz CMA and Mirmiran M (1996). Hypothalamic Integration of Circadian Rhythms.

[B20] Swaab DF (1995). Development of the human hypothalamus. Neurochem Res.

[B21] Swaab DF, Van Someren EJ, Zhou JN, Hofman MA (1996). Biological rhythms in the human life cycle and their relationship to functional changes in the suprachiasmatic nucleus. Prog Brain Res.

[B22] Swaab DF, Hofman MA, Lucassen PJ, Purba JS, Raadsheer FC, Van de Nes JA (1993). Functional neuroanatomy and neuropathology of the human hypothalamus. Anat Embryol (Berl).

[B23] Swaab DF, Hofman MA (1994). Age, sex and light: variability in the human suprachiasmatic nucleus in relation to its functions. Prog Brain Res.

[B24] Kalamatianos T, Kallo I, Coen CW (2004). Ageing and the diurnal expression of the mRNAs for vasopressin and for the V1a and V1b vasopressin receptors in the suprachiasmatic nucleus of male rats. J Neuroendocrinol.

[B25] Weinert H, Weinert D, Schurov I, Hastings MH, Maywood ES (2001). Impaired expression of the mPer2 circadian clock gene in the suprachiasmatic nuclei of aging mice. Chronobiol Int.

[B26] Reiter RJ, Richardson BA (1992). Some perturbations that disturb the circadian melatonin rhythm. Chronobiol Int.

[B27] Siegmund R, Tittel M, Schiefenhovel W (1998). Activity Monitoring of the inhabitants of Tauwema, a traditional Melanesian village: rest/activity behavior of Trobriand Islands Papua New Guinea. Biologiacl Rhythm Research.

[B28] van Coevorden A, Mockel J, Laurent E, Kerkhofs M, L'Hermite-Balériaux M, Decoster C, Nève P, Van Cauter E (1991). Neuroendocrine rhythms and sleep in aging men. Am J Physiol.

[B29] Kripke DF, Elliott JA, Youngstedt SD, Rex KM (2007). Circadian phase response curves to light in older and young women and men. J Circadian Rhythms.

[B30] Duffy JF, Dijk DJ, Klerman EB, Czeisler CA (1998). Later endogenous circadian temperature nadir relative to an earlier wake time in older people. Am J Physiol.

[B31] Campbell SS, Murphy PJ (1998). Relationships between sleep and body temperature in middle-aged and older subjects.. J Am Geriatr Soc.

[B32] Winn B, Whitaker D, Elliott DB, Phillips NJ (1994). Factors affecting light-adapted pupil size in normal human subjects. Invest Ophthalmol Vis Sci.

[B33] Brainard GC, Gaddy JR, Ruberg FM, Barker FM, Hanifin JP, Rollag MD, Moller M and Pevet P (1994). Ocular mechanisms that regulate the human pineal gland.

[B34] TC J, RT L, Y B, SW J, RH M (2005). Retinal ganglion cell degeneration is topological but not cell type specific in DBA/2J mice.. J Cell Biol.

[B35] Blumenthal EZ, Weinreb RN (2001). Assessment of the retinal nerve fiber layer in clinical trials of glaucoma neuroprotection. Surv Ophthalmol.

[B36] Haefliger IO, Fleischhauer JC, Flammer J (2000). In glaucoma, should enthusiasm about neuroprotection be tempered by the experience obtained in other neurodegenerative disorders?. Eye.

[B37] Zhang Y, Kornhauser JM, Zee PC, Mayo KE, Takahashi JS, Turek FW (1996). Effects of aging on light-induced phase-shifting of circadian behavioral rhythms, Fos expression and CREB phosphorylation in the hamster suprachiasmatic nucleus. Neurosci.

[B38] Witting W, Mirmiran M, Bos NP, Swaab DF (1993). Effect of light intensity on diurnal sleep-wake distribution in young and old rats. Brain Res Bull.

[B39] Turek FW, Penev P, Zhang Y, Reeth OV, Takahashi JS, Zee P (1995). Alterations in the circadian system in advanced age. Ciba Foundation Symposium.

[B40] Leger D, Guilleminault C, Defrance R, Domont A, Paillard M (1999). Prevalence of sleep/wake disorders in persons with blindness. Clin Sci (Colch).

[B41] Zizi F, Jean-Louis G, Magai C, Greenidge KC, Wolintz AH, Heath-Phillip O (2002). Sleep complaints and visual impairment among older Americans: a community-based study. J Gerontol A Biol Sci Med Sci.

[B42] Jean-Louis G, Kripke DF, Ancoli-Israel S, Klauber M, Sepulveda RS, Mowen MA, Assmus JD, Langer RD (2000). Circadian sleep, illumination, and activity patterns in women: Influences of aging and time reference.. Physiol Behav.

[B43] Campbell SS, Kripke DF, Gillin JC, Hrubovcak JC (1988). Exposure to light in healthy elderly subjects and Alzheimer's patients. Physiol Behav.

[B44] Kripke DF, Jean-Louis G, Elliott JA, Klauber MR, Rex KM, Tuunainen A, Langer RD (2004). Ethnicity, sleep, mood, and illumination in postmenopausal women.. BMC Psychiatry.

[B45] Van Someren EJ, Kessler A, Mirmiran M, Swaab DF (1997). Indirect bright light improves circadian rest-activity rhythm disturbances in demented patients. J Biol Rhythms.

[B46] Kripke DF, Youngstedt SD, Elliott JA (1998). Possible light resistance of aging Americans. SLTBR Abstracts.

[B47] Loving RT, Kripke DF, Knickerbocker NC, Grandner MA (2005). Bright green light treatment of depression for older adults [ISRCTN69400161]. BMC Psychiatry.

[B48] Jean-Louis G, Kripke D, Cohen C, Zizi F, Wolintz A (2005). Associations of Ambient Illumination With Mood: Contribution of Ophthalmic Dysfunctions.. Physiol Behav.

[B49] Jean-Louis G, Kripke D, Elliot JA, Zizi F, Wolintz A, Lazzaro DR (2005). Daily Illumination Exposure and Melatonin: Influence of Ophthalmic Dysfunctions and Sleep Duration. J Cir Rhythm.

[B50] Sack RL, Lewy AJ, Blood ML, Keith LD, Nakagawa H (1992). Circadian rhythm abnormalities in totally blind people: Incidence and clinical significance. J Clin Endocrinol Metab.

[B51] Tabandeh H, Lockley SW, Buttery R, Skene DJ, Defrance R, Arendt J, Bird AC (1998). Disturbance of sleep in blindness. Am J Ophthalmol.

[B52] Jongebloed WL, Kalicharan D, Los LI, Worst JG (1993). The Morgagnian and Brunescens cataract morphology studied with with SEM and TEM. Eur J Morphol.

[B53] Klein R, Moss SE, Klein BE (1987). New management concepts for timely diagnosis of diabetic retinopathy treatable by photocoagulation. Diabetes Care.

[B54] VanNewkirk MR, Nanjan MB, Wang JJ, Mitchell P, Taylor HR, McCarty CA (2000). The prevalence of age-related maculopathy: the visual impairment project. Ophthalmology.

[B55] van Soest S, Westerveld A, de Jong PT, Bleeker-Wagemakers EM, Bergen AA (1999). Retinitis pigmentosa: defined from a molecular point of view. Surv Ophthalmol.

[B56] Kripke DF (1998). Light treatment for nonseasonal depression: speed, efficacy, and combined treatment. J Affect Dis.

[B57] Sommer A, Tielsch JM, Katz J, Quigley HA, Gottsch JD, Javitt JC, Martone JF, Royall RM, Witt KA, Ezrine S (1991). Racial differences in the cause-specific prevalence of blindness in east Baltimore. N Engl J Med.

[B58] Leske MC, Connell AM, Wu SY, Nemesure B, Li X, Schachat A, Hennis A (2001). Incidence of open-angle glaucoma: the Barbados Eye Studies. The Barbados Eye Studies Group. Arch Ophthalmol.

[B59] Cermakian N, Sassone-Corsi P (2002). Environmental stimulus perception and control of circadian clocks. Curr Opin Neurobiol.

[B60] Provencio I, Rodriguez IR, Jiang G, Hayes WP, Moreira EF, Rollag MD (2000). A novel human opsin in the inner retina. J Neurosci.

[B61] Lucas RJ, Douglas RH, Foster RG (2001). Characterization of an ocular photopigment capable of driving pupillary constriction in mice. Nat Neurosci.

[B62] Liu JH (1998). Circadian rhythm of intraocular pressure. J Glaucoma.

[B63] Leske MC, Connell AM, Wu SY, Hyman L, Schachat AP (1997). Distribution of intraocular pressure. The Barbados Eye Study. Arch Ophthalmol.

[B64] Harris A, Rechtman E, Siesky B, Jonescu-Cuypers C, McCranor L, Garzozi HJ (2005). The role of optic nerve blood flow in the pathogenesis of glaucoma. Ophthalmol Clin North Am.

[B65] Frampton P, Da Rin D, Brown B (1987). Diurnal variation of intraocular pressure and the overriding effects of sleep. Am J Optom Physiol Opt.

[B66] Flammer J, Orgul S, Costa VP, Orzalesi N, Krieglstein GK, Serra LM, Renard JP, Stefansson E (2002). The impact of ocular blood flow in glaucoma. Prog Retin Eye Res.

[B67] Harris A, Kagemann L, Cioffi GA (1998). Assessment of human ocular hemodynamics. Surv Ophthalmol.

[B68] Grieshaber MC, Flammer J (2005). Blood flow in glaucoma. Curr Opin Ophthalmol.

[B69] Chung HS, Harris A, Evans DW, Kagemann L, Garzozi HJ, Martin B (1999). Vascular aspects in the pathophysiology of glaucomatous optic neuropathy. Surv Ophthalmol.

[B70] Gherghel D, Orgul S, Gugleta K, Gekkieva M, Flammer J (2000). Relationship between ocular perfusion pressure and retrobulbar blood flow in patients with glaucoma with progressive damage. Am J Ophthalmol.

[B71] Gherghel D, Hosking SL, Orgul S (2004). Autonomic nervous system, circadian rhythms, and primary open-angle glaucoma. Surv Ophthalmol.

[B72] Kato M, Roberts-Thomson P, Phillips BG, Haynes WG, Winnicki M, Accurso V, Somers VK (2000). Impairment of endothelium-dependent vasodilation of resistance vessels in patients with obstructive sleep apnea. Circulation.

[B73] Mojon A, Hess CW, Goldblum D, Boehnke M, Koerner F, Gugger M, Bassetti C, Mathis J (2002). Normal-tension glaucoma is associated with sleep apnea syndrome.. Ophthalmologica.

[B74] Jean-Louis G, Kripke D, Zizi F, Lazarro D, Wolintz A, Tsai J, Harris A (2005). Retinal Blood Flow Is Associated With The Phase Of Endogenous Melatonin. Sleep.

[B75] Buysse DJ, Monk TH, Reynolds CFII, Jarrett DB, Jennings RJ, Hoch CC, Kupfer DJ, Kuna ST, Suratt PM and Remmers JE (1991). Circadian rhythms in the healthy elderly. Sleep and Respiration in Aging Adults.

[B76] Eastman CI, Boulos Z, Terman M, Campbell SS, Dijk DJ, Lewy AJ (1995). Light treatment for sleep disorders: consensus report. VI. Shift work. J Biol Rhythms.

[B77] Richardson GS, Malin HV (1996). Circadian rhythm sleep disorders: pathophysiology and treatment. J Clin Neurophysiol.

[B78] Regestein QR, Widiger TA, Frances AJ, Pincus HA, First MB, Ross R and Davis W (1994). Circadian rhythm sleep disorder (Sleep-wake schedule disorder). DSM-IV Sourcebook.

[B79] Shibui K, Uchiyama M, Okawa M (1999). Melatonin rhythms in delayed sleep phase syndrome. J Biol Rhythms.

[B80] Rosenwasser AM, Wirz-Justice A, Redfern P and Lemmer B (1997). Circadian rhythms and depression :  clinical and experimental models.. Handbook of Experimental Pharmacology: Vol 125.

[B81] Casten RJ, Rovner BW, Tasman W (2004). Age-related macular degeneration and depression: a review of recent research. Curr Opin Ophthalmol.

[B82] Heikkinen RL, Kauppinen M (2004). Depressive symptoms in late life: a 10-year follow-up. Arch Gerontol Geriatr.

[B83] Tsai SY, Cheng CY, Hsu WM, Su TP, Liu JH, Chou P (2003). Association between visual impairment and depression in the elderly. J Formos Med Assoc.

[B84] Lamberg L (1998). Blind people often sleep poorly; research shines light on therapy. JAMA.

[B85] Wee R, Van Gelder RN (2004). Sleep disturbances in young subjects with visual dysfunction. Ophthalmology.

[B86] Klerman EB, Rimmer DW, Dijk DJ, Kronauer RE, Rizzo JF, Czeisler CA (1998). Nonphotic entrainment of the human circadian pacemaker. Am J Physiol.

[B87] Czeisler CA, Shanahan TL, Klerman EB, Martens H, Brotman DJ, Emens JS, Klein T, Rizzo JF (1995). Suppression of melatonin secretion in some blind patients by exposure to bright light [see comments]. N Engl J Med.

[B88] Czeisler CA (1995). The effect of light on the human circadian pacemaker. Ciba Found Symp.

[B89] Gaddy JR, Ruberg FL, Brainard GC, Rollag MD, Jung EG and Holick MF (1994). Pupillary modulation of light-induced melatonin suppression. Biologic effects of light.

[B90] Hattar S, Lucas RJ, Mrosovsky N, Thompson S, Douglas RH, Hankins MW, Lem J, Biel M, Hofmann F, Foster RG, Yau KW (2003). Melanopsin and rod-cone photoreceptive systems account for all major accessory visual functions in mice. Nature.

[B91] Berson DM, Dunn FA, Takao M (2002). Phototransduction by retinal ganglion cells that set the circadian clock. Science.

[B92] Hattar S, Liao HW, Takao M, Berson DM, Yau KW (2002). Melanopsin-containing retinal ganglion cells: architecture, projections, and intrinsic photosensitivity. Science.

[B93] Van Gelder RN (2003). Making (a) sense of non-visual ocular photoreception. Trends Neurosci.

[B94] Sekaran S, Lupi D, Jones SL, Sheely CJ, Hattar S, Yau KW, Lucas RJ, Foster RG, Hankins MW (2005). Melanopsin-dependent photoreception provides earliest light detection in the mammalian retina. Curr Biol.

[B95] Panda S, Nayak SK, Campo B, Walker JR, Hogenesch JB, Jegla T (2005). Illumination of the melanopsin signaling pathway. Science.

[B96] Sancar A (2000). Cryptochrome: the second photoactive pigment in the eye and its role in circadian photoreception. Annu Rev Biochem.

[B97] Perez-Leon JA, Warren EJ, Allen CN, Robinson DW, Lane Brown R (2006). Synaptic inputs to retinal ganglion cells that set the circadian clock.. Eur J Neurosci.

[B98] J, J H, Fahrenkrug, J (2007). Synaptic Contact between Melanopsin-Containing Retinal Ganglion Cells and Rod Bipolar Cells.. Invest Ophthalmol Vis Sci.

[B99] Newman LA, Walker MT, Brown RL, Cronin TW, Robinson PR (2003). Melanopsin forms a functional short-wavelength photopigment. Biochemistry.

[B100] Hannibal J, Hindersson P, Knudsen SM, Georg B, Fahrenkrug J (2002). The photopigment melanopsin is exclusively present in pituitary adenylate cyclase-activating polypeptide-containing retinal ganglion cells of the retinohypothalamic tract. J Neurosci.

[B101] Gooley JJ, Lu J, Fischer D, Saper CB (2003). A broad role for melanopsin in nonvisual photoreception. J Neurosci.

[B102] Panda S, Provencio I, Tu DC, Pires SS, Rollag MD, Castrucci AM, Pletcher MT, Sato TK, Wiltshire T, Andahazy M, Kay SA, Van Gelder RN, Hogenesch JB (2003). Melanopsin is required for non-image-forming photic responses in blind mice. Science.

[B103] Lucas RJ, Foster RG (1999). Photoentrainment in mammals: a role for cryptochrome?. J Biol Rhythms.

[B104] Czeisler CA, Kronauer RE, Mooney JJ, Anderson JL, Allan JS (1993). Biologic rhythm disorders, depression, and phototherapy. A new hypothesis. Int J Neurosci.

[B105] Hannibal J, Hindersson P, Ostergaard J, Georg B, Hegge FW (2004). Melanopsin is expressed in PACAp containing retinal ganglion cells of the human retinohypothalamic tract.. Investigative Ophthalmology and Visual Science.

[B106] Frishman LJ, Shen FF, Du L, Robson JG, Harwerth RS, Smith EL, Carter-Dawson L, Crawford ML (1996). The scotopic electroretinogram of macaque after retinal ganglion cell loss from experimental glaucoma. Invest Ophthalmol Vis Sci.

[B107] Bui BV, Fortune B (2004). Ganglion cell contributions to the rat full-field electroretinogram. J Physiol.

[B108] Brzezinski JA, Brown NL, Tanikawa A, Bush RA, Sieving PA, Vitaterna MH, Takahashi JS, Glaser T (2005). Loss of circadian photoentrainment and abnormal retinal electrophysiology in Math5 mutant mice. Invest Ophthalmol Vis Sci.

[B109] RS L, DK T, HH C, ML P, KF S (2006). Melanopsin-expressing retinal ganglion cells are more injury-resistant in a chronic ocular hypertension model.. Invest Ophthalmol Vis Sci.

[B110] (2002). National Eye Institute Vision Report.  Vision Problems in the U.S.: Prevalence of adult vision impairment and age-related eye disease in America (2002). http://www.nei.nih.gov/eyedata/pdf/VPUS.pdf.

